# The utility of two interview-based physical activity questionnaires in healthy young adults: Comparison with accelerometer data

**DOI:** 10.1371/journal.pone.0203525

**Published:** 2018-09-07

**Authors:** René Schilling, Eveline Schärli, Xenia Fischer, Lars Donath, Oliver Faude, Serge Brand, Uwe Pühse, Lukas Zahner, Simon Rosenbaum, Philip B. Ward, Attilio Carraro, Markus Gerber

**Affiliations:** 1 Department of Sport, Exercise and Health, University of Basel, Basel, Switzerland; 2 Department of Intervention Research in Exercise Training, German Sport University, Cologne, Germany; 3 Psychiatric Clinic of the University of Basel, Basel, Switzerland; 4 School of Psychiatry, UNSW, Sydney, Australia; 5 Black Dog Institute, Randwick, Australia; 6 Schizophrenia Research Unit, Ingham Institute of Applied Medical Research, Liverpool, Australia; 7 Department of Biomedical Sciences, University of Padua, Padua, Italy; Vanderbilt University, UNITED STATES

## Abstract

**Background:**

Accurate assessment of physical activity is essential to determine the magnitude of the health-related benefits of regular physical activity. While physical activity questionnaires are easy to use, their accuracy in comparison to objective measures has been questioned. The purpose of the present study was to examine the utility of two interview-based questionnaires; a recently-developed instrument, the Simple Physical Activity Questionnaire (SIMPAQ), and the Seven Day-Physical Activity Recall (7DPAR).

**Methods:**

Accelerometer data was collected in 72 university students (50% females). Telephone interviews were conducted to complete the SIMPAQ and the 7DPAR.

**Results:**

Significant correlations (*p* < .001) were found between accelerometer-based moderate-to-vigorous physical activity (MVPA), the amount of self-reported moderate-to-vigorous exercise assessed via the SIMPAQ (*rho* = .49), and vigorous physical activity assessed via the 7DPAR (*rho* = .50). Exercise assessed via the SIMPAQ was significantly correlated with the vigorous physical activity score of the 7DPAR (*rho* = .56, *p* < .001). While participants needed three minutes less to complete the SIMPAQ (*p* < .001), participants tended to be more confident about the accuracy of the answers they provided on the 7DPAR (*p* < .01).

**Conclusions:**

These two questionnaire measures of physical activity performed similarly in a healthy young adult sample. The SIMPAQ can be completed in 15 minutes, which could be an advantage in settings where time for physical activity assessment is limited.

## Background

To document the health-related benefits of regular physical activity and the health risks associated with a physically inactive lifestyle [1], it is essential for sports, exercise and health scientists to accurately assess physical activity [2, 3]. Reliable and validated tools are essential to measure prevalence rates, estimate risk ratios, monitor changes in physical activity levels, and to formulate public health recommendations for specific target groups [2, 4].

Although some researchers argued that doubly labeled water technique and accelerometers should be seen as the gold standard to assess physical activity [3, 5, 6], doubly labeled water only provides information about energy expenditure, without providing information about time spent in physical activity at different intensity levels or in different contexts. Moreover, this technique is expensive and complex, and thus not suited for epidemiological research or clinical settings, where information must often be obtained in a short timeframe to allow clinical recommendations to be made to patients [6]. While accelerometers are less expensive and more practical, they are unable to accurately measure activities such as rowing, biking, and weight lifting [6, 7]. Although combined with heart rate, accelerometers can provide more detailed information about physical activity participation, they are still not commonly used in clinical practice [7]. Most likely this is due to relatively high costs, the burden placed on the participants (because the device must be worn for several days), issues associated with compliance, and the need for skilled personnel to analyze the data [7, 8].

Physical activity questionnaires are easy to use, do not require significant financial investments from the investigator, do not require significant motivation or time from the participant, and data is immediately available [7]. This may explain why self-report measures are widely used in research and clinical practice [2]. Nevertheless, some questions have been raised about the validity of physical activity questionnaires [2, 3, 7]. Existing self-report instruments, such as the International Physical Activity Questionnaire (IPAQ), were developed for population surveillance, and in clinical settings may be considered too long to complete, or may lead to overestimates of participants’ physical activity levels [9, 10]. Correlations with objective measures have frequently been found to be relatively modest, although statistically significant in the large scale studies in which they are typically evaluated [11]. Another widely used instrument, the Seven-Day Physical Activity Recall (7DPAR), does not assess sedentary behavior, and correlations with accelerometer data were moderate for most measures obtained and also may both over- or underestimate physical activity levels [11–13]. A brief, yet comprehensive instrument to assess physical activity behavior, the Simple Physical Activity Questionnaire (SIMPAQ) [10], was developed with the aim of more accurately assessing physical activity. The current exploratory study was undertaken to compare this new tool with existing questionnaires, and an objective measure of physical activity in a convenience sample of healthy young adults attending university to study exercise and health science. We examined the relationships between questionnaire measures of physical activity and the same measures obtained via accelerometry, and evaluated the inter-correlations between questionnaire measures. We also evaluated the time taken to complete each questionnaire, and subjective perceptions of the accuracy and ease of completing these measures.

## Methods

### Participants

Participants were recruited at the Department of Sport, Exercise and Health of the University of Basel, Switzerland. Power analysis (using G*Power 3.1 software) showed that at least 64 participants were needed to detect (one-tailed) correlations of *r* ≥.30 (alpha error: .05, power: .80) [14]. After having received detailed oral and written information about the study, 83 exercise and health science students (40 men, 43 women; 67 undergraduate, 16 graduate) provided written informed consent. One student dropped out, seven students had to be excluded because of technical reasons (accelerometer malfunction), and one accelerometer was lost. Furthermore, two students had to be excluded due to insufficient accelerometer wear-time (see below for more information). Thus, the final sample consisted of 72 students (36 men, 36 women).

### Procedures

Students were informed about the study during a lecture in the spring term (April to May 2016). After providing informed consent, students who wished to take part in the study provided information about anthropometry and demographic background including gender, age, weight, height, living situation, relationship status, and health status. Participants were given an accelerometer (ActiGraph^®^ wGT3X-BT, Actigraph, Pensacola, USA), and were instructed to wear the device for a minimum of seven and a maximum of 14 days. Instructions on the use of the accelerometer and non-wear time sheets were handed out to each participant to systematically document periods when they did not wear the accelerometer. They were asked to provide times for a telephone interview. The telephone interview included the Simple Physical Activity Questionnaire (SIMPAQ) [10] and the Seven Day-Physical Activity Recall (7DPAR) [15], and took place seven to 14 days after the accelerometer has been distributed. Random assignment was used to ensure that 50% of the participants first completed the SIMPAQ or the 7DPAR, respectively. Additionally, participants rated the intelligibility and convenience of the instruments, and provided an estimate about their confidence in the accuracy of their answers. Both the SIMPAQ and the 7DPAR referred to the previous seven days.

Prior to the data collection, the study protocol was approved by the local ethical review board (Ethics Committee of Northwestern and Central Switzerland: EKNZ; approval number: 2016/272), and the study was carried out in accordance with the ethical principles described in the Declaration of Helsinki. As an incentive, all students took part in a prize draw for ten cinema tickets (worth approximately 13 Euro each).

### Measures

#### Accelerometry

Physical activity was objectively assessed with the ActiGraph^®^ wGT3X-BT accelerometer, a light triaxial activity monitor. Evidence of the validity and reliability of this instrument to accurately capture physical activity has been documented previously [6].

The monitor was placed on a strap around the wrist of the non-dominant hand, and participants were asked to wear it continuously during the study period, except for activities taking place in water lasting longer than 30 minutes or taking place below one meter of water surface. Non-wear periods were reported on a non-wear time sheet.

The sample rate was 30 Hz. Recordings were saved as raw data files and analyzed with the ActiLife Software (Version 6.13.2). Data were summed and stored in 60 seconds epoch lengths. Daily summed minute-by-minute data were categorized by cut-off values. ActiLife software does not provide validated cut-off values for wrist worn devices in adults. Therefore, we applied the recently developed cut-off values by Kamada et al. [16], which were <2000 vector magnitude counts per minute (VM cpm) for sedentary, 2000 to 7499 VM cpm for light physical activity, and ≥7500 VM cpm for moderate-to-vigorous physical activities [16]. In their validation study, Kamada et al. showed, in a sample of 94 US adults, that these cutoffs are best suited to minimize the mean differences of hip vs. wrist worn accelerometry (using the same instrument as we did in our study) over a 7-day measurement period. Physical activities listed on the non-wear time sheet were included as moderate physical activity if they also were identified as non-wear time by the ActiLife software [17]. Non-wear time was determined using the Troiano [18] algorithm with default settings. Following Clemente et al. [19], days with ≥10 percent of non-wear time were considered non-valid and excluded. To be included, participants had to have at least five valid days, including ≥4 valid weekdays and ≥1 valid weekend day (only considering the 7 days prior to the telephone interview). Sleep was calculated using the Cole-Kripke [20] algorithm with sleep period detection options provided by ActiGraph^®^. Weekly scores were obtained by dividing the sum of all valid days through the number of valid days, and then multiplying by seven. The following indices were examined (in min per week): sleep, sedentary activity, light physical activity (LPA) and moderate-to-vigorous physical activity (MVPA). Moreover, the weekly number of steps was measured.

#### Simple Physical Activity Questionnaire (SIMPAQ)

The SIMPAQ is a brief five-item tool, which comprehensively evaluates activity over the past seven days including time in bed, sedentary time, time spent walking, type and time spent in exercise, and time spent for other activities [10]. Assessed physical activity refers to all domains of activity, including leisure time, domestic, work and transport-related activities. The SIMPAQ captures a 24-hour period representative for the previous week. An additional sixth item (time spent standing) was added to the SIMPAQ in the present study, based on experience in this healthy young adult group obtained during pilot testing of the SIMPAQ, which took place before the official start of the data assessment and was carried out with three staff members of the Department of Sport, Exercise and Health at the University of Basel.

To ensure optimal translation, we rigorously followed the procedure set out by Brislin [21]. English items were translated into German, and then back-translated into English by an independent translator (see supporting information S1 Fig for the wording of German items, see www.simpaq.org for the English version of the instrument). The following indices were generated (in min/week): time in bed, sedentary time, time spent standing, time spent walking, other physical activities, and exercise.

#### Seven-Day Physical Activity Recall (7DPAR)

The 7DPAR is a widely-used instrument to assess physical activity [13, 15]. Evidence regarding the validity and reliability of this instrument has been reported previously [13, 15]. The 7DPAR assesses physical activity day-by-day for the previous seven days. Participants are asked to first report time spent in bed and then time for physical activities with ≥10 min duration and at least moderate intensity (in the morning, afternoon and evening) [15]. The 7DPAR starts with the previous day, and then refers to prior preceding days. Participants were asked to classify the intensity of the reported activities into moderate, hard or very hard. In the following, hard and very hard intensity were considered as vigorous and very vigorous in the interest of uniformity and clarity. The interviewers also assessed breaks during the activities, which are then subtracted from total activity time. For each day, additional information about time spent in strength and flexibility training is assessed. The 7DPAR provides information about the following seven parameters (in min/week): sleep, moderate physical activity (MPA), vigorous physical activity (VPA), strength training, and flexibility training.

### Statistical analyses

All analyses were conducted with SPSS Statistics 23 for Windows (IBM Corporation, Armonk, USA). Throughout all analyses, the level of significance was set at *p*≤0.05. First, descriptive statistics were calculated to describe the characteristics of the sample and the level of self-reported and objectively assessed physical activity. Normality was tested with Kolmogorov-Smirnov test. As not all parameters were distributed normally, Spearman correlation analyses were computed to study pairwise associations between indicators of self-reported and objectively assessed physical activity (all referring to the 7 days prior to the telephone interview). Spearman correlations were also used to examine bivariate relationships between the SIMPAQ and 7DPAR variables. Repeated measures analyses of variance (rANOVAs) with one within-subject factor (SIMPAQ vs. 7DPAR) were used to find out whether mean scores in time necessary to complete the SIMPAQ and 7DPAR and their usability differed from each other. Univariate outliers, defined at ≥3 standard deviations from the mean, were identified, resulting in one outlier for the time needed to complete the SIMPAQ. Because exclusion of the outlier did not substantially influence the results, this case was included in the further statistical analyses. Following the recommendations of Cohen [22], correlations of *rho* < .30 were considered small, with *rho* = .30 to .50 as medium and *rho* >.50 as large.

## Results

### Sample characteristics

Of the 72 participants included in the analyses, 36 answered the SIMPAQ first, and 36 answered the 7DPAR first. Males and females were both equally represented in the sample (36 each), with 18 males and 18 females first completing the SIMPAQ or 7DPAR, respectively. Most of the participants reported that they are physically healthy (*n* = 69, 96%), whereas 3 participants (4%) indicated that they currently had an injury, which prevented them from running for 15 minutes. Fifty-six participants (78%) were undergraduate students, 16 (22%) were master’s students. The average age of the participants was 22.6±2.2 years, ranging from 19 to 29 years. The mean height and body weight were 180.1±6.5 cm and 77.3±8.2 kg for men, and 168.9±5.8 and 62.1±4.6 for women, respectively. The mean body mass index (BMI) was 22.5±2.3 kg/m^2^ and the mean number of steps was 14075±2879 per day.

### Descriptive results for physical activity

Descriptive statistics for self-reported physical activity and accelerometer-based data are presented in Table 1. When the six activities assessed in the SIMPAQ were summed up (sleep, sedentary, standing, walking, other activities, exercise), the mean score was 23.3±1.4 hours (range: 17.3 to 25.2 hours), which corresponds well with the target 24-hour period.

**Table 1:** Descriptive statistics for anthropometric measures and physical activity.

**Variable**	***M***	***SD***	**Range**	**Skewness**	**Kurtosis**	**K-S**
Age in years	22.5	2.5	19–29	1.12	0.91	0.16***
Height (cm; all participants)	174.5	9.4	157–193	0.13	-0.84	0.11*
Men	180.1	6.5	167–193	-0.28	-0.28	0.13
Women	168.9	5.8	157–180	0.38	0.42	0.20
Weight (kg; all participants)	68.7	11.2	52–90	0.42	-0.97	0.15**
Men	75.3	8.2	61–90	-0.07	-0.70	0.20
Women	62.1	4.6	52–75	0.72	1.84	0.09
BMI (kg*m^-2^)	22.5	2.3	18.02–29.05	0.73	1.04	0.06
**SIMPAQ**	***M***	***SD***	**Range**	**Skewness**	**Kurtosis**	**K-S**
Time in Bed (min/week)	3479.1	446.2	2310.0–5355.0	0.67	3.82	0.14**
Sedentary Time (min/week)	3369.9	953.8	605.0–5775.0	-0.33	0.55	0.08
Time Spent Standing (min/week)	962.5	569.5	140.0–2730.0	1.02	1.08	0.18***
Time Spent Walking (min/week)	795.5	603.2	70.0–3255.0	1.83	4.20	0.19***
Other Physical Activities (min/week)	365.1	306.4	0.0–1575.0	1.56	2.68	0.24***
Exercise (min/week)	775.6	414.2	0.0–2070.0	0.65	0.43	0.08
**7DPAR**	***M***	***SD***	**Range**	**Skewness**	**Kurtosis**	**K-S**
Sleep (min/week)	3375.5	345.0	2100.0–4830.0	0.39	5.06	0.07
MPA (min/week)	461.0	337.4	0.0–1530.0	1.10	0.74	0.14**
VPA (min/week)	499.9	348.6	0.0–1515.0	0.73	-0.06	0.10*
Strength Training (min/week)	75.8	83.8	0.0–360.0	1.58	1.98	0.21***
Flexibility Training (min/week)	27.7	42.5	0.0–260.0	2.87	11.83	0.26***
**Accelerometer**	***M***	***SD***	**Range**	**Skewness**	**Kurtosis**	**K-S**
Sleep (min/week)	3131.2	321.8	2044.0–3937.0	-0.26	1.39	0.08
Sedentary Time (min/week)	4177.9	627.7	3140.8–6210.5	0.93	1.16	0.09
LPA (min/week)	2601.0	389.7	1573.0–3777.0	0.18	0.69	0.07
MVPA (min/week)	653.7	277.6	228.0–1565.0	0.91	0.91	0.10
Steps (number/week)	98527.4	20149.9	15345.0–139006.0	-0.97	3.04	0.09

*Notes*: kg = kilograms. m = meters. min = minutes. MPA = Moderate physical activity. VPA = Vigorous physical activity. LPA = Light physical activity. MVPA = Moderate-to-vigorous physical activity. SIMPAQ = Simple Physical Activity Questionnaire. 7DPAR = Seven Day Physical Activity Recall. Accelerometer: ActiGraph^®^ WgT-3X. K-S = Kolmogorov-Smirnov Test.

*p < .05.

**p < .01.

***p < .001.

### Correlations between accelerometer data and self-reported physical activity

#### Simple Physical Activity Questionnaire (SIMPAQ)

Spearman correlation coefficients were calculated between the SIMPAQ and accelerometer data. As shown in Table 2, relatively hgh correlations were found between self-reported exercise and objectively assessed MVPA (*rho* = .49, *p* < .001) and number of steps (*rho* = .56, *p* < .001). Moreover, weak-to-moderate positive correlations were found for self-reported and objectively assessed sedentary time (*rho* = .26, *p* < .05) and time spent in bed/sleep (*rho* = .35, *p* < .01).

**Table 2:** Spearman correlations between subjective measures and accelerometer data for all participants (*N* = 72).

Variable	Accelerometer				
	Sleep	Sedentary Time	LPA	MVPA	Steps
**SIMPAQ**					
Time in Bed (min/week)	.35**	-.02	-.01	-.06	-.25*
Sedentary Time (min/week)	-.07	.26*	-.31**	-.29*	-.46***
Time Spent Standing (min/week)	-.03	-.08	.09	.02	.03
Time Spent Walking (min/week)	-.13	.05	.19	.00	.29*
Other Physical Activities (min/week)	-.14	.11	-.02	-.02	.25*
Exercise (min/week)	-.06	-.29*	.06	.49***	.56***
**7DPAR**					
Sleep (min/week)	.48***	-.08	-.13	-.06	-.17
MPA (min/week)	-.12	-.22	.34**	.12	.21
VPA (min/week)	.10	-.28*	.11	.50***	.54***
Strength Training (min/week)	.00	-.10	.04	.09	-.01
Flexibility Training (min/week)	-.13	-.09	.21	.26*	.12

*Notes*: min = minutes. MPA = Moderate physical activity. VPA = Vigorous physical activity. LPA = Light physical activity. MVPA = Moderate-to-vigorous physical activity. SIMPAQ = Simple Physical Activity Questionnaire. 7DPAR = Seven Day Physical Activity Recall. Accelerometer: ActiGraph^®^ WgT-3X.

*p < .05.

**p < .01.

***p < .001

#### Seven-Day Physical Activity Recall (7DPAR)

Relatively high correlations were found between self-reported vigorous physical activity and accelerometer-based MVPA (*rho* = .50*p* < .001) and number of steps (*rho* = .54, *p* < .001). A moderate-to-strong correlation was found between objectively and self-reported sleep (*rho* = .48, *p* < .001)

### Correlations between self-report physical activity questionnaires

Spearman correlations were also computed between the measures derived from the SIMPAQ and the 7DPAR. Results are presented in Table 3. Most importantly, a relatively strong correlation was found between the exercise score of the SIMPAQ and the VPA score of the 7DPAR (*rho* = .56, *p* < .001).

**Table 3:** Spearman correlations between subjective measures.

**Variable**	**1**.	**2**.	**3**.	**4**.	**5**.	**6**.	**7**.	**8**.	**9**.	**10**.
**SIMPAQ**										
1. Time in Bed (min/week)	-									
2. Sedentary Time (min/week)	-.18	-								
3. Time Spent Standing (min/week)	-.16	-.19	-							
4. Time Spent Walking (min/week)	-.13	-.40**	.10	-						
5. Other Physical Activities (min/week)	-.17	-.19	-.07	.25*	-					
6. Exercise (min/week)	-.21	-.15	-.07	-.07	.01	-				
**7DPAR**										
7. Sleep (min/week)	.46***	-.10	-.05	.00	-.18	-.11	-			
8. MPA (min/week)	-.01	-.16	.14	.28*	.08	.07	.08	-		
9. VPA (min/week)	-.09	-.32**	-.25*	.03	.15	.56***	-.08	-.08	-	
10. Strength Training (min/week)	.05	-.03	-.20	-.10	.11	.08	.01	-.01	.17	-
11. Flexibility Training (min/week)	-.09	-.07	-.16	-.16	.10	.25*	-.12	-.03	.13	.18

*Notes*: min = minutes. MPA = Moderate physical activity. VPA = Vigorous physical activity. MVPA = Moderate-to-vigorous physical activity. SIMPAQ = Simple Physical Activity Questionnaire. 7DPAR = Seven Day Physical Activity Recall. Accelerometer: ActiGraph^®^ WgT-3X.

*p < .05.

**p < .01.

***p < .001.

### Usability of the SIMPAQ and 7DPAR

As shown in Table 4, significantly less time was needed to complete the SIMPAQ than the 7DPAR (15.0 min ± 6.1 vs. 18.1 min ± 6.1, *p* < .001). Participants rated the 7DPAR as slightly easier to complete than the SIMPAQ (*p* < .05), and participants reported higher confidence in the accuracy of their answers when answering the 7DPAR (*p* < .01).

**Table 4:** Comparison of the usability of the SIMPAQ and 7DPAR.

Variable	Overall (*N* = 72)		SIMPAQ first (*n* = 36)		7DPAR first (*n* = 36)		Instrument			Order of administration			Instrument x Order of administration		
	*M*	*SD*	*M*	*SD*	*M*	*SD*	*F*	*p*	η^2^	*F*	*p*	η^2^	*F*	*p*	η^2^
Time needed to complete the SIMPAQ	15.0	6.1	16.4	7.3	13.6	4.2	32.41	.000	.32	1.48	.228	.02	5.24	.025	.07
Time needed to complete the 7DPAR	18.1	6.1	18.3	6.0	17.9	6.3									
Intelligibility of SIMPAQ items	9.0	1.4	9.3	1.2	8.7	1.5	1.70	.197	.02	1.54	.219	.02	3.80	.055	.05
Intelligibility of 7DPAR items	9.1	1.1	9.2	1.2	9.1	1.1									
Convenience of SIMPAQ items	5.3	2.1	5.4	2.2	5.3	2.0	6.15	.016	.08	.02	.888	.00	.14	.713	.00
Convenience of 7DPAR items	5.8	1.6	5.8	1.7	5.8	1.5									
Confidence in accuracy of answers for SIMPAQ	6.3	1.6	6.4	1.4	6.1	1.8	11.75	.001	.15	.56	.458	.01	9.72	.003	.12
Confidence in accuracy of answers for 7DPAR	6.9	1.4	6.5	1.4	7.3	1.3									

*Notes*: SIMPAQ = Simple Physical Activity Questionnaire. 7DPAR = Seven Day Physical Activity Recall.

When participants first completed the 7DPAR, confidence in the accuracy of their answers was higher for the 7DPAR compared to the SIMPAQ (*p* < .01), whereas participants needed more time to complete the 7DPAR than the SIMPAQ (*p* < .001). When participants first completed the SIMPAQ, participants still needed less time to complete the SIMPAQ than the 7DPAR (*p* < .05).

## Discussion

This study compared measures of physical activity obtained with two interview-based questionnaires in healthy young adults. The recently developed SIMPAQ was found to have adequate measurement properties and exercise-based physical activity derived from this tool was moderately to strongly correlated with objective accelerometer data. The SIMPAQ exercise score also correlated with VPA assessed via a previously well-validated self-report questionnaire (7DPAR). We also found that the SIMPAQ was completed in less time than the 7DPAR. Participants felt more confident in the accuracy of their answers when answering the 7DPAR. However, confidence in the accuracy of the answers only differed if participants had completed the 7DPAR first.

One of the novel features of the SIMPAQ is a focus on assessing all activities over a 24-hour period. In the current study, participants reported an average of 23 hours and 20 minutes of activities, suggesting a high degree of accuracy in estimating activity typical of a 24-hour period. We included an additional question to capture time spent standing, as in this healthy young adult population we found a relatively large amount of time was spent standing each day (approximately 2 hours and 20 minutes). Future research will be needed to establish whether this additional question would be equally useful in more sedentary or clinical populations.

Exercise assessed via the SIMPAQ was moderately to strongly associated with accelerometer-based data, with correlations ranging from *rho* = .49 (MVPA) to *rho* = .56 (number of steps). Thus, compared to a systematic review, in which Helmerhorst et al. [14] found Spearman correlations of *rho*≈.30 in adults across several studies, stronger evidence for the criterion-validity of the two self-report questionnaires was found in the present sample. Several reasons exist why it is difficult to detect even stronger correlations between self-reported and objectively assessed physical activity. While accelerometry is a relatively feasible, and accurate method to assess physical activity [6], accelerometers measure the acceleration of the worn sensor, and therefore sometimes lead to misinterpretations with regard to particular types of physical activity. Some established limitations are the underestimation of walking and overestimation of jogging, the failure to detect resistance exercise and external work, and bicycle riding or the fact that the accelerometer must be taken off during some sport activities (e.g. swimming, during soccer games) [23]. Although speculative, the fact that no significant correlations were found for time spent walking and other physical activities with accelerometer-based MVPA might suggest that these activities do not reach moderate-intensity levels. Nevertheless, both walking and other physical activities were correlated with total number of accelerometer-based steps.

Similar correlations were observed for accelerometer data with the SIMPAQ and the 7DPAR, suggesting that criterion validity was acceptable for both interview-based instruments. Our findings are partly in line with prior research showing reasonably high correlations between the 7DPAR variables and accelerometer-based data, including indicators such as total energy expenditure, or time in MVPA [12, 13]. The finding that the MPA score of the 7DPAR was only weakly correlated with total accelerometer-based MVPA might be attributable to the fact that some self-reported physical activities might not have been captured as such by the Kamada et al. [16] algorithm for wrist-worn accelerometer data. Since few established algorithms exist for wrist-worn accelerometer data [24], we also used the more established algorithm of Freedson et al. [25] for hip-worn accelerometer data to examine our data. As shown in the supporting information (S1 Table), the correlations between the two questionnaires and the accelerometer data are similar (or slightly higher) for both the SIMPAQ and the 7DPAR. Data in S2 Table in the supporting information also shows reasonably high associations between the accelerometer scores for sedentary activity, MPA, VPA and MVPA, if data based on the Kamada et al. [16] and Freedson et al. [25] algorithm were correlated.

The SIMPAQ completion time was shorter than the 7DPAR, making it more efficient for both the investigator and the interviewee. While no remarkable differences were found for intelligibility and convenience, confidence in the accuracy of the given answers was higher for the 7DPAR. Although speculative, we assume that the latter finding is due to the characteristics of this sample. Thus, because exercise and health science students attend multiple exercise lessons throughout the week, using a day-to-day format as offered by the 7DPAR might increase their confidence that they accurately remembered all activities. Future research is needed to examine economy and usability of the SIMPAQ in less active samples who have lower levels of regular exercise participation.

In the present sample, accelerometer-based MVPA amounted to 654 min/week, while the amount of moderate-to-vigorous exercise assessed via the SIMPAQ was 776 min/week and the amount of VPA assessed via the 7DPAR 500 min/week. However, the question of which questionnaire more accurately estimates physical activity is difficult to answer because the SIMPAQ and 7DPAR contain different dimensions. In line with this notion, previous research was inconclusive whether physical activity assessed via interviews leads to an over- or underestimation of physical activity levels. For instance, a systematic review of studies validating the International Physical Activity Questionnaire [11] found incorrect estimates of total MVPA ranging from -28% to +173% for studies using accelerometry. Similarly, a recently published validation study of the 7DPAR [13] reported approximately 20% overestimation of MVPA. The reason why the self-report questionnaires can differ from the accelerometer data are multifold, including recall bias, incorrect perceptions of intensity of physical activity, interviewer effects, social desirability, limited compliance in wearing accelerometer devices, or problems of accelerometers to accurately assess all physical activities [26]. These issues notwithstanding, the present study showed that the SIMPAQ is relatively well-suited to capture activities across a 24-hours period, which might lead to more realistic physical activity estimates compared to other self-report instruments.

The strengths of this study were that questionnaires were applied in a counter-balanced order during the telephone interview, an equal number of men and women was included, relatively strict non-wear time limitations were applied, and dropout and exclusion rates were low.

Despite these advantages, the present study has certain limitations. First, the sample consisted of highly active, generally healthy university students, which is a particular group in relation to their commitment towards physical activity and sport. Although the SIMPAQ can be used in any population, the main idea behind the development of the SIMPAQ was to provide a simple self-report measure to assess physical activity in populations at high risk of sedentary behavior, such as people with psychiatric disorders [10]. Therefore, the validity of the SIMPAQ needs to be established in these specific target groups. Second, as reported above, the correlations between the SIMPAQ and 7DPAR may be positively biased, because the instruments were completed sequentially. Third, to increase compliance, participants were asked to wear accelerometers on the wrist [27]. While some argued that the wrist-worn accelerometers lead to less accurate (mostly lower) estimates of physical activity compared to waist- or back-worn devices [28, 29], others found only minor differences due to wear locations [27, 30], or indicated that sleep detection is inaccurate if the devices are worn around the hip [31, 32]. Specifically, McMinn et al. [28] found that the selection of the ‘worn on wrist’ option leads to a systematic underestimation of energy expenditure. We therefore decided not to select the ‘worn on wrist’ option when analyzing the data. Fourth, another possible limitation associated with the scoring of the accelerometers is related to the epoch length. Although not systematically studied in adults, research on children indicates that an epoch length of 60 seconds might underestimate participants’ VPA counts [29].

## Conclusions

The present study found that two interview-based questionnaire measures of physical activity resulted in similar findings in healthy young adults. The recently developed SIMPAQ proved to take three minutes less to complete than the 7DPAR, which could be an advantage in settings where time for physical activity assessment is limited. Research is currently underway to validate the SIMPAQ in more sedentary populations.

## Supporting information

S1 Fig Wording of the German version of the SIMPAQ used in the present study. (DOCX)

S1 Table Spearman correlations between subjective measures and accelerometer data. (DOCX)

S2 Table Bivariate correlations between accelerometer data using the Freedson et al. or Kamada et al. algorithm. (DOCX)

## Abbreviations

7DPAR: Seven Day Physical Activity Recall
LPA: Light physical activity
MET: Metabolic equivalent of task
MPA: Moderate physical activity
MVPA: Moderate-to-vigorous physical activity
SIMPAQ: Simple Physical Activity Questionnaire
TST: Total sleep time
VPA: Vigorous physical activity
