# Supplementary material for: The utility of two interview-based physical activity questionnaires in healthy young adults: Comparison with accelerometer data
Source: PLoS One. 2018 Sep 7;13(9):e0203525. doi: 10.1371/journal.pone.0203525 (PMC6128548; doi:10.1371/journal.pone.0203525)
Supplement: S1 Fig — (DOCX) [file pone.0203525.s001.docx]

**Supporting Information**

The utility of two physical activity questionnaires in healthy young adults:

Comparison with accelerometer data

S1 Figure provides wording of the German version of the SIMPAQ used in the present study.

**SIMPAQ**

**Einleitung:** Ich frage Dich nun danach, welche Aktivitäten Du während den **letzten sieben Tagen** wie lange getan hast Dazu gehört die Zeit, die Du im Bett verbracht hast, während der Du gesessen oder gelegen hast, während der Du gestanden bist, während der Du gelaufen bist, während der Du in der Freizeit oder im Beruf anderweitig körperlich aktiv warst, oder während der Du trainiert oder Sport getrieben hast.

1A. Um welche Zeit bist Du während den letzten sieben Tagen meistens zu Bett gegangen?

Hinweis: zwischen ______ und ______ Uhr?

**Antwort:** _______ Uhr

1. Durchschnittliche Anzahl Stunden pro Nacht im Bett:

1B. Um welche Zeit bist Du während den letzten sieben Tagen meistens aufgestanden?

**Antwort:** _______ Uhr

2A. Damit bleiben rund _______ Stunden pro Tag, die Du nicht im Bett verbracht hast. Wie viele dieser _______ Stunden hast Du mit Sitzen oder Liegen verbracht, z.B. während Du gegessen, gelesen, TV geschaut oder elektronische Geräte verwendet hast (wie Handy, Tablet, Computer)?

Hinweis: z.B. Sitzen während der Arbeit, beim Pendeln oder beim Autofahren, während der Freizeit oder zuhause?

2. Durchschnittliche Anzahl Stunden pro Tag sitzend/liegend:

**Antwort:** _____ Stunden und _____ Minuten (pro Tag)

2B. Wie viel von dieser Zeit hast Du geschlummert bzw. gedöst?

**Antwort:** _____ Stunden und _____ Minuten (pro Tag)

3. Damit bleiben rund _______Stunden pro Tag, die Du nicht im Bett oder sitzend und liegend verbracht hast. Denke nun an alle stehenden Aktivitäten, die Du als Teil Deiner beruflichen Tätigkeit oder zuhause (z.B. Kochen, Zähne putzen, duschen) ausführst. Wie viele Minuten hast Du pro Tag an den meisten Tagen der letzten Woche stehend verbracht? Hinweis: Nicht eingeschlossen sind darin zu Fuss gehen (Frage 4), Sport, sonstiges Training (Frage 6) oder sonstige berufliche oder Freizeitaktivitäten (Frage 5).

3. Durchschnittliche Anzahl Stunden pro Tag für stehende Aktivitäten:

**Antwort:** ___ Stunden und ___ Minuten (pro Tag)

4. Damit bleiben rund _______Stunden pro Tag für andere Aktivitäten. An welchen Tagen während der letzten Woche bist Du zu Fuss gegangen, um zu trainieren, zu spazieren, wandern, oder von einem Ort zum anderen zu gelangen? Wie viele Minuten bist Du an den einzelnen Tagen ungefähr zu Fuss gegangen?

4. Durchschnittliche Anzahl Stunden pro Tag gehend:

| Montag | Dienstag | Mittwoch | Donnerstag | Freitag | Samstag | Sonntag |
| --- | --- | --- | --- | --- | --- | --- |
|  |  |  |  |  |  |  |

5. Denke nun an alle weiteren körperlichen Aktivitäten, die Du als Teil Deiner beruflichen Tätigkeit oder zuhause (z.B. Gartenarbeit, Arbeiten im Haushalt) ausführst (Kein Sport). Wie viele Minuten warst Du pro Tag im Rahmen dieser Tätigkeiten an den meisten Tagen der letzten Woche aktiv? Hinweis: Nicht eingeschlossen sind darin zu Fuss gehen (Frage 4), Sport oder sonstiges Training (Frage 6)

5. Durchschnittliche Anzahl Stunden pro Tag für andere körperliche Aktivitäten:

**Antwort:** ___ Stunden und ___ Minuten (pro Tag)

6A. Denke nun an alle weiteren Aktivitäten, die Du tust, um zu trainieren oder Sport zu treiben wie Jogging, Schwimmen, Fahrradfahren, Yoga, Training im Fitnesscenter, praktische Vorlesung oder Unisport. An welchen Tagen der letzten Woche hast Du solche Aktivitäten gemacht?

Hinweis: Nicht eingeschlossen ist zu Fuss gehen (z.B. zügiges Gehen, Spazieren, Wandern). Diese Aktivitäten werden in Frage 4 erfasst.

6B. Welche Aktivitäten hast Du genau gemacht? Mit welcher Intensität und wie lange hast Du diese Aktivitäten an den einzelnen Tagen gemacht?

|  | **Aktivität und Intensität (0-10)** | | **Anzahl Trainingseinheiten** | **Minuten** | **Total** |
| --- | --- | --- | --- | --- | --- |
| *z.B.* | Krafttraining (5/10); Tennis (9/10) | | 1; 1 | 15; 50 | 65 |
| Montag |  | |  |  |  |
| Dienstag |  | |  |  |  |
| Mittwoch |  | |  |  |  |
| Donnerstag |  | |  |  |  |
| Freitag |  | |  |  |  |
| Samstag |  | |  |  |  |
| Sonntag |  | |  |  |  |
|  | | **Total** |  |  |  |

4. Durchschnittliche Anzahl Stunden pro Tag für Sport/Training:

von 24

**Stunden Gesamt:**

**S1 Figure. Wording of the German version of the SIMPAQ used in the present study.**
