# Supplementary material for: The utility of two interview-based physical activity questionnaires in healthy young adults: Comparison with accelerometer data
Source: PLoS One. 2018 Sep 7;13(9):e0203525. doi: 10.1371/journal.pone.0203525 (PMC6128548; doi:10.1371/journal.pone.0203525)
Supplement: S1 Table — (DOCX) [file pone.0203525.s002.docx]

**Supporting Information**

The utility of two physical activity questionnaires in healthy young adults:

Comparison with accelerometer data

S1 Table provides the bivariate correlations between subjective measures of physical activity assessed via the SIMPAQ/7DPAR and the accelerometer data using the Freedson et al. [1] instead of Kamada et al. [2] algorithm.

**S1 Table. Spearman correlations between subjective measures and accelerometer data.**

| (*N*=72) | Accelerometer (Freedson et al. algorithm) | | | | | | |
| --- | --- | --- | --- | --- | --- | --- | --- |
| SIMPAQ | Sleep | Sedentary | LPA | MPA | VPA | MVPA | Steps |
| Time in Bed (min/week) | .35** | -.03 | -.05 | -.12 | -.13 | -.18 | -.25* |
| Sedentary Time (min/week) | -.07 | .28* | .03 | -.36** | -.28* | -.35** | -.46*** |
| Time Spent Standing (min/week) | -.03 | -.04 | -.09 | .17 | .03 | .11 | .03 |
| Time Spent Walking (min/week) | -.13 | .07 | -.13 | .23 | .05 | .20 | .29* |
| Other Physical Activities (min/week) | -.14 | .08 | -.10 | .12 | .05 | .11 | .25* |
| Exercise (min/week) | -.06 | -.15 | -.29* | .20 | .58*** | .41*** | .56*** |
| 7DPAR |  |  |  |  |  |  |  |
| Sleep (min/week) | .48*** | -.09 | -.09 | -.20 | -.13 | -.23 | -.17 |
| MPA (min/week) | -.12 | -.17 | -.04 | .37** | .12 | .31** | .21 |
| VPA (min/week) | .10 | -.20 | -.28* | .28* | .57*** | .45*** | .54*** |
| Strength Training (min/week) | .00 | -.18 | .00 | .02 | -.03 | .01 | -.01 |
| Flexibility Training (min/week) | -.13 | -.14 | .07 | .12 | .12 | .17 | .12 |

*Notes:* min=minutes. LPA=Light physical activity. MPA=Moderate physical activity. VPA=Vigorous physical activity. MVPA=Moderate-to-vigorous physical activity. SIMPAQ=Simple Physical Activity Questionnaire. 7DPAR=Seven Day Physical Activity Recall.

Cut-off values of counts per minute for physical activity levels were ≤1951 for light, 1952-5724 for moderate, ≥5724 for vigorous physical activities (1).

**p*<.05. ***p*<.01. ****p*<.001

**References**

1. Freedson PS, Melanson E, Sirard J. Calibration of the Computer Science and Applications, Inc. accelerometer. Med Sci Sports Exerc. 1998;30: 777-781.

2. Kamada M, Shiroma EJ, Harris TB, Lee I. Comparison of physical activity assessed using hip- and wrist-worn accelerometers. Gait Posture. 2016;44: 23-28.
