# Supplementary material for: The utility of two interview-based physical activity questionnaires in healthy young adults: Comparison with accelerometer data
Source: PLoS One. 2018 Sep 7;13(9):e0203525. doi: 10.1371/journal.pone.0203525 (PMC6128548; doi:10.1371/journal.pone.0203525)
Supplement: S2 Table — (DOCX) [file pone.0203525.s003.docx]

**Supporting Information**

The utility of two physical activity questionnaires in healthy young adults:

Comparison with accelerometer data

S2 Table provides the bivariate correlations between accelerometer data using the Freedson et al. [1] or Kamada et al. [2] algorithm.

**S2 Table. Bivariate correlations between accelerometer data using the Freedson et al. or Kamada et al. algorithm.**

| (*N*=72) | Accelerometer (Freedson et al. algorithm) | | | | |
| --- | --- | --- | --- | --- | --- |
| Accelerometer  (Kamada et al. algorithm) | Sedentary | LPA | MPA | VPA | MVPA |
| Sedentary | .85*** | .07 | -.56*** | -.48*** | -.41*** |
| LPA | -.53*** | .31*** | .79*** | .19 | .51*** |
| MVPA | -.53*** | -.25* | .51*** | .89*** | .65*** |

*Notes:* min=minutes. LPA=Light physical activity. MPA=Moderate physical activity. VPA=Vigorous physical activity. MVPA=Moderate-to-vigorous physical activity.

**p*<.05. ***p*<.01. ****p*<.001

**References**

1. Freedson PS, Melanson E, Sirard J. Calibration of the Computer Science and Applications, Inc. accelerometer. Med Sci Sports Exerc. 1998;30: 777-781.

2. Kamada M, Shiroma EJ, Harris TB, Lee I. Comparison of physical activity assessed using hip- and wrist-worn accelerometers. Gait Posture. 2016;44: 23-28.
